# Measuring disability-adjusted life years (DALYs) due to COVID-19 in Scotland, 2020

**DOI:** 10.1186/s13690-022-00862-x

**Published:** 2022-04-01

**Authors:** Grant M. A. Wyper, Eilidh Fletcher, Ian Grant, Gerry McCartney, Colin Fischbacher, Oliver Harding, Hannah Jones, Maria Teresa de Haro Moro, Niko Speybroeck, Brecht Devleesschauwer, Diane L. Stockton

**Affiliations:** 1 Place and Wellbeing Directorate, Public Health Scotland, Glasgow, Scotland; 2 Data Driven Innovation Directorate, Public Health Scotland, Edinburgh, Scotland; 3 College of Social Sciences, University of Glasgow, Glasgow, Scotland; 4 Clinical and Protecting Health Directorate, Public Health Scotland, Edinburgh, Scotland; 5 Directorate of Public Health, NHS Forth Valley, Stirling, Scotland; 6 Research Institute of Health and Society (IRSS), Université catholique de Louvain, Brussels, Belgium; 7 Department of Epidemiology and Public Health, Sciensano, Brussels, Belgium; 8 Department of Translational Physiology, Infectiology and Public Health, Ghent University, Merelbeke, Belgium

**Keywords:** Burden of disease, disability-adjusted life years, DALY, YLD, YLL, population health, Scottish burden of disease, European burden of disease network, coronavirus, COVID-19

## Abstract

**Background:**

Disability-adjusted life years (DALYs) combine the impact of morbidity and mortality and can enable comprehensive, and comparable, assessments of direct and indirect health harms due to COVID-19. Our aim was to estimate DALYs directly due to COVID-19 in Scotland, during 2020; and contextualise its population impact relative to other causes of disease and injury.

**Methods:**

National deaths and daily case data were used. Deaths were based on underlying and contributory causes recorded on death certificates. We calculated DALYs based on the COVID-19 consensus model and methods outlined by the European Burden of Disease Network. DALYs were presented as a range, using a sensitivity analysis based on Years of Life Lost estimates using: cause-specific; and COVID-19 related deaths. All COVID-19 estimates were for 2020.

**Results:**

In 2020, estimates of COVID-19 DALYs in Scotland ranged from 96,500 to 108,200. Direct COVID-19 DALYs were substantial enough to be framed as the second leading cause of disease and injury, with only ischaemic heart disease having a larger impact on population health. Mortality contributed 98% of total DALYs.

**Conclusions:**

The direct population health impact of COVID-19 has been very substantial. Despite unprecedented mitigation efforts, COVID-19 developed from a single identified case in early 2020 to a condition with an impact in Scotland second only to ischaemic heart disease. Periodic estimation of DALYs during 2021, and beyond, will provide indications of the impact of DALYs averted due to the national rollout of the vaccination programme and other continued mitigation efforts, although new variants may pose significant challenges.

**Supplementary Information:**

The online version contains supplementary material available at 10.1186/s13690-022-00862-x.

## Background

The World Health Organization (WHO) declared the outbreak of novel coronavirus disease (COVID-19) a public health emergency of international concern (PHEIC) on 30th January 2020 [1]. It was subsequently designated as a global pandemic on 11th March 2020 by the WHO Director General [2]. Early in the pandemic, the largest share of worldwide deaths emerged in Europe, with recorded estimates of over half-a-million COVID-19 deaths in Europe during 2020 [3]. In Scotland, the first confirmed case was detected in Tayside on 1st March 2020, in a person who had recently returned from travel in Northern Italy [4]. Since then, COVID-19 has had a devastating impact in Scotland in 2020 – around 127,000 positive cases were identified, and over 6,800 COVID-19 related deaths were recorded, with deaths occurring most frequently in the elderly, vulnerable and frail [5, 6].

Many efforts are being made to assess the direct impact of COVID-19, including publication of daily numbers of suspected and confirmed positive cases, hospitalisations and hospitalisations requiring intensive care. In Scotland, both the Scottish Government and National Records of Scotland (NRS) have reported on the extent of COVID-19 mortality using different definitions [5, 6]. All of this information contributes to building an understanding of the direct population impact of COVID-19. Scottish and United Kingdom (UK) wide studies have quantified the comparative fatal population health impact through the loss of life expectancy at birth, years of life lost to premature mortality (YLL) and quality-adjusted life years (QALYs) [7–9]. Studies in other countries have combined the impact of morbidity and mortality of COVID-19 in a consistent and comparable way using disability-adjusted life years (DALYs), to allow framing in the wider context of other causes of disease and injury [10, 11]. To date, COVID-19 DALY studies have not yet been carried out in the UK, or any of its constituent nations.

Burden of disease assessments allow estimates of the occurrence of morbidity and mortality to be translated into a single measure facilitating comparisons with other causes of disease and injury [12]. This is achieved by standardising the effects of morbidity and mortality as population health loss as a function of time, using the composite measure of DALYs [13]. The occurrence of morbidity is translated to estimates of years lived with disability (YLD) by adjusting for severity and relative disability suffered due to each cause of disease or injury. Mortality counts are translated into estimates of YLL, using aspirational or national age-conditional life tables, recognising that deaths at younger ages have a greater impact on population health. DALYs are estimated by summing YLD and YLL. As causes of disease or injury are measured in a consistent way using DALYs, disease burden studies have become an increasingly popular way to assess population health impact as a means to support and influence national, and local, decision-making. The Scottish Burden of Disease study (SBOD) was set up to comprehensively estimate the population health impact of causes of disease and injury through estimating DALYs using routine data sources [14]. Integration of estimates of COVID-19 DALYs is, therefore, essential to ensure that this assessment remains comprehensive across all causes of disease and injury.

The aim of this study was to estimate the direct impact of COVID-19 on the population health of Scotland during 2020; the first full calendar year in which COVID-19 was a globally recognised disease entity, and to contextualise its population impact relative to other causes of disease and injury.

## Methods

### Data

Data on death registrations was sourced from NRS [6]. Scottish death registration data is perceived as high-quality and has a low level of recorded ill-defined deaths [15]. Death data were provisional, as final mortality statistics are updated based on results of legal enquiries into some deaths. This mainly affects deaths from external causes, including suicide and drug-related deaths. COVID-19 deaths were identified as those where the underlying, or any contributory, cause of death was coded using the emergency ICD-10 codes U07.1 or U07.2, based on guidance from the WHO [16]. Deaths were included in this study if the date of death was during 2020, with the first death occurring in mid-March 2020. The dataset was generated on 25th February 2021, to allow additional time to capture the later registrations of some deaths occurring towards the end of 2020.

Case estimates were sourced from the Public Health Scotland (PHS) SEIR (Susceptible → Exposed → Infected → Recovered) transmission model [16]. The PHS SEIR model is a stochastic compartmental model used for real-time monitoring and forecasting of key COVID-19 epidemiological estimates. The model incorporates several streams of real-world Scottish data on reported cases, testing intensity, hospitalisations, critical care occupancy and deaths. The model assumptions and approach have been described in detail elsewhere, and the model builds upon work used in response to the 2009-2010 influenza epidemic in Malta [17, 18]. Estimates from this model were used to calculate person-years of disability for community-based (moderate) cases, and to identify the total number of people that were infected. Person-years take into account the number of people that were infected and the duration of time that each person suffered health loss, and therefore avoids the need for any assumption about assigning average durations to the number of infections. Symptomatic infections were characterised in three ways: (i) community-based, not requiring hospitalisation; (ii) requiring hospitalisation but not intensive care unit admission; and, (iii) requiring intensive care. Daily data from PHS were used to calculate the person-years due to hospitalised and intensive care unit cases [5]. Estimates from earlier in the pandemic were based on both suspected and confirmed cases. However from 22^nd^ July 2020, data are based on confirmed cases only as suspected cases were no longer published. We opted to include suspected cases to reflect that reported confirmed cases were likely to be biased by the lack of polymerase chain reaction testing in earlier months.

The most recent estimates of the number of DALYs due to other causes of disease and injury in 2018 were estimated from the SBOD study, to allow the number of DALYs due to COVID-19 to be framed within a ranking of causes of health loss prior to the pandemic (Table S1) [14]. Additionally, estimates of health loss in Scotland derived by the Global Burden of Disease (GBD) 2019 study were extracted from the GBD Results Tool for 2019 by individual cause [19].

### Analyses

The modelling approach to calculating DALYs was based on the COVID-19 consensus model and methods outlined by the European Burden of Disease Network and the European Centre for Disease Prevention and Control (ECDC) [20].

Mortality counts were aggregated by five-year age-group and sex to calculate YLL. For the purposes of YLL calculations, the under-5 years age-group was split into under 1 year and 1 to 4 years, and the upper open-ended age-group was set at 95 years and above. YLL estimates were derived by multiplying the number of deaths in each age-group by the age-conditional life expectancy defined by the GBD 2019 reference life table, which assigns the same values to both males and females [21].

Daily data, on the number of COVID-19 cases, were used to compute the number of person-years for each COVID-19 health state. Person-years were calculated as the sum of the number of symptomatic cases reported each day, scaled by a factor of $\frac{1}{365.25}$ (to reflect the contribution of individual days to a complete year, since DALYs use year as the unit of time). To estimate YLD due to post-acute consequences for “long-COVID” patients, we assumed that approximately 1-in-7 people (13.3%) that were infected would suffer post-acute consequences for four weeks (28 days), reflecting early available transition probabilities [22]. Health states were defined using descriptions and disability weights from the GBD 2019 and European Disability Weight Study (EDWS) and are shown in Table 1 [24, 25]. Person-years were multiplied by disability weights for each health state to estimate prevalence-based YLD. For the acute health states, person-years were estimated on a daily basis therefore we did not require any estimate of the duration of cases. YLD were summed with YLL to estimate DALYs.

**Table 1:** COVID-19 health states, attributable disability weights and data sources

Health state name	Health state description	Data source	Disability Weight
Asymptomatic	Non-hospitalised community infection: Has infection but experiences no symptoms	PHS SEIR transmission model [17, 18, 23]	Nil
Infectious disease: acute episode, moderate	Non-hospitalised community infection: Has a fever and aches, and feels weak, which causes some difficulty with daily activities.	PHS SEIR transmission model, GBD 2019 [17, 18, 23, 24]	0.051
Infectious disease: acute episode, severe	Hospitalised infection: Has a high fever and pain, and feels very weak, which causes great difficulty with daily activities.	PHS, GBD 2019 [5, 24]	0.133
Infectious disease: acute episode, intensive care	Hospitalised infection, requiring intensive care unit admission with or without respiratory support	PHS, EDWS [5, 25]	0.655
Infectious disease: post-acute consequences	Suffers from symptomatic health loss post infection, such as fatigue, emotional lability, or insomnia	Based on published transition probabilities of total number of cases (1-in-7), with 28-day duration [22, 24]	0.219

‘PHS’ denotes Public Health Scotland’ ‘SEIR’ denotes Susceptible → Exposed → Infected → Recovered; ‘GBD’ denotes Global Burden of Disease; ‘EDWS’ denotes European Disability Weight Study

Estimates were framed in terms of ranking of causes of DALYs using a pre-COVID-19 pandemic counterfactual ranking of the leading causes of disease or injury in 2018. All COVID-19 estimates presented are for calendar year 2020 in Scotland.

### Uncertainty and sensitivity analyses

The greatest sources of uncertainty in our estimates relate to biases in the measurement of the number of cases – particularly community infections – and the proportion of cases that suffer from post-acute consequences of COVID-19, rather than random variation in our data systems. Thus, we firstly used univariate sensitivity analyses to quantify the impact around these uncertainties (Table S2). Sensitivity analyses related to uncertainty around the number of community cases were based on increasing the number of moderate cases by a fixed percentage, similar to the method adopted in another study of COVID-19 DALYs [10]. Our sensitivity analyses based on the transition from acute to post-acute consequences from infection, were based on applying different transition probabilities in two ways, for: (i) all cases; and, (ii) symptomatic cases only. We also varied our assumptions by providing scenarios with half or double the duration of post-acute consequences. Finally, we included two multivariate sensitivity analyses which were based on the results of the univariate sensitivity analyses. These were designed to maximise, and minimise, the YLD impact of all combined univariate scenarios.

For mortality, we outlined two scenarios and have presented our main estimate of DALYs as a range based on this sensitivity. This range does not reflect a confidence interval and should not be interpreted as such. This range is based on using cause-specific deaths (i.e. where the underlying cause of death was COVID-19) and YLL calculated using deaths involving COVID-19 (i.e. where either the underlying, or any of up to 10 contributing causes mentioned on the death certificate was COVID-19).

When framing our main estimated range of DALYs, we performed an additional sensitivity analysis using the most recent pre-pandemic estimates extracted from GBD 2019 (Table S3). This was to illustrate whether the framing of COVID-19 differed based on whether SBOD or GBD estimates were used.

## Results

### Direct population health impact of COVID-19

**Fig. 1:**
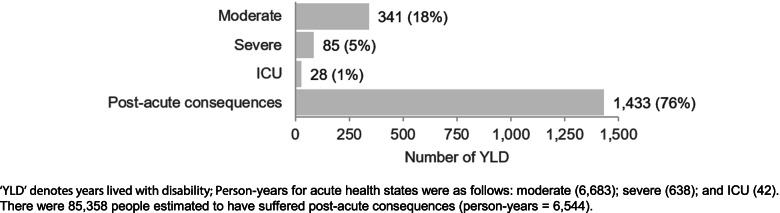
Estimates of COVID-19 YLD by health state, Scotland, 2020

It was estimated that 641,789 people were infected by COVID-19 in Scotland in 2020 (Table 2). Taking into account the duration of infection, we estimated this reflected 7,363 person-years infected with symptomatic COVID-19 (90.8% moderate; 8.7% severe; and, 0.6% intensive care unit (ICU)) which generated 453 YLD from acute infection (Fig. 1). It was estimated that approximately 85,400 people suffered from post-acute consequences, such as fatigue and emotional lability, which generated a further 1,433 YLD, giving 1,886 YLD directly due to acute and post-acute consequences of COVID-19. Around three-quarters of YLD (76% of total YLD) was generated from post-acute consequences of COVID-19. Although ICU cases were the most debilitating to individuals, their short-term population impact on total YLD was the smallest (1% of total YLD) because of the relatively small number of patients who required ICU treatment.

**Table 2:** Summary of measures of direct COVID-19 population health impact, Scotland, 2020

Metric		Number
Morbidity	Persons	641,789
	Person-years (symptomatic)	7,363
	YLD	1,886
Mortality	Deaths	6,167 – 6,845
	YLL	94,633 – 106,357
DALYs		96,519 – 108,243

‘YLD’ denotes years lived with disability; ‘YLL’ denotes years of life lost to premature mortality; ‘DALYs’ denotes disability-adjusted life years; estimates representative for calendar year 2020

In 2020, there were 6,845 COVID-19 related deaths, of which 6,167 were cause-specific deaths, with YLL estimates of 106,357 and 94,633, respectively. YLL per COVID-19 death ranged from 15.3 to 15.5 YLL. Overall it was estimated that in 2020, DALYs directly due to COVID-19 were between 96,519 and 108,243. The majority of DALYs were generated through the impact of mortality, with YLL representing 98% of DALYs.

**Fig. 2:**
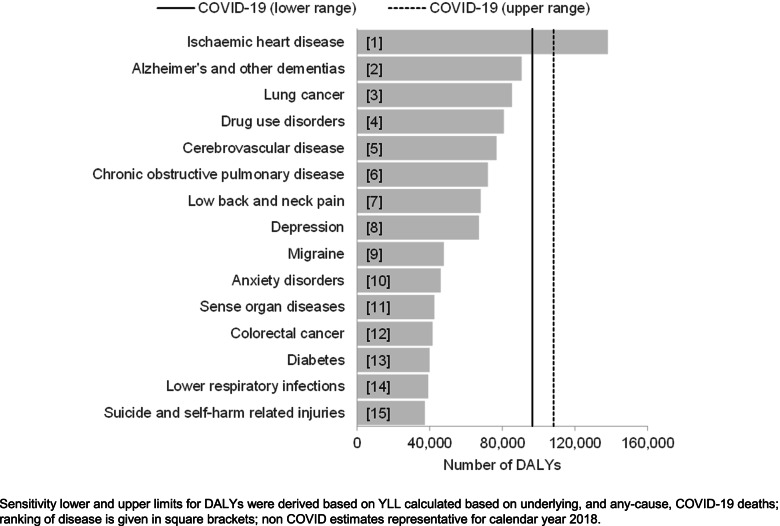
Estimates of the number of DALYs for COVID-19 and the pre-pandemic (2018) 15 leading causes of disease/injury, Scotland, 2020

When comparing the number of direct COVID-19 DALYs with the pre-COVID-19 leading causes of disease and injury in Scotland, COVID-19 generated enough DALYs in 2020 to represent the second leading cause of death and disability in Scotland (Fig. 2). The estimated lower limit of DALYs was higher than DALYs from Alzheimer’s disease and other dementias, whereas the estimated upper limit of DALYs was considerably less than the DALYs due to ischaemic heart disease.

### Sensitivity analyses

Through combinations of assumptions designed to minimise, or maximise, the impact on YLD estimates, our estimates of YLD ranged from 632 to 6,356 YLD (Table S2). These were largely driven by assumptions over the incidence and duration of post-acute consequences, as undercounts of milder cases did not have a large impact on overall YLD estimates. None of our defined scenarios changed our finding that COVID-19 DALYs likely represented the second leading cause of death and disability in Scotland in 2020. This is because YLD only contributes around 2% of total COVID-19 DALYs.

Ranking our range of direct COVID-19 DALYs (including incorporating results from our sensitivity analyses) using the alternative GBD 2019 Scottish results indicated that COVID-19 would have represented the second leading cause of disease in Scotland (Table S3). This is consistent with the framing in our main analyses using SBOD estimates, with DALYs falling short of those due to ischaemic heart disease, but higher than all other individual causes of disease and injury.

## Discussion

### Summary

We estimated that there were between 96,500 to 108,200 DALYs directly due to COVID-19 in Scotland during 2020. DALYs due to COVID-19 were likely to have had the second largest population health impact, relative to all other individual causes of disease and injury. The combined impact of COVID-19 morbidity and mortality was lower than ischaemic heart disease, but higher than all other leading causes of DALYs, such as: Alzheimer's and other dementias, lung cancer, and drug use disorders. The majority of population health loss was due to mortality, representing 98% of DALYs, and 15 YLL were estimated per COVID-19 death.

These conclusions remain consistent across our full range of sensitivity analyses, which were designed to accommodate a range of scenarios regarding: potential under-ascertainment of community (non-hospitalised) infections; and the transition to, and duration of, post-acute consequences from acute COVID-19 infection. The framing of COVID-19 as the second leading cause was also consistent in our sensitivity analysis using findings for Scotland from the GBD 2019 study.

Although individual COVID-19 cases requiring hospital, or intensive care support, have considerable consequences for the ill-health of individuals, post-acute consequences following infection (such as fatigue or emotional lability) pose the largest cumulative impact on population health when measured by YLD for survivors. Although evidence on the risk and duration of post-acute consequences is highly uncertain, this conclusion remains consistent throughout our wide range of sensitivity analyses. However, caution should remain over interpreting health state differences in YLD in such a crude way, given that many hospitalisations (particularly those requiring intensive care) would have had the highest mortality risks and therefore have expected to result in YLL.

### Strengths and weaknesses

Although our estimates of COVID-19 mortality are based on provisional death registrations, they are unlikely to significantly differ from final mortality estimates. The largest differences between provisional and final cause of death estimates are in external and ill-defined causes. During the early emergence of COVID-19 there was potential for deaths not to be certified as COVID-19, particularly in the absence of a positive test, but it is difficult to quantify any bias related to under- or over-recording of COVID-19 deaths [26]. Some burden of disease studies attempt to propagate the uncertainty around DALY estimates by estimating uncertainty intervals, which combine sampling error and non-sampling errors arising from study design. This approach was developed to avoid misleading users over how precise confidence intervals were, given they only capture the extent of sampling error. However, other approaches have been developed to give a sense of how uncertain an estimate might be. We have chosen not to estimate uncertainty intervals, as we believe that by presenting our estimates as a range, we have incorporated the main factors which are likely to have the largest impact on our estimates.

When framing the number of DALYs within a pre-pandemic cause list, there are some potential pitfalls to consider, related to competing risk of death. It is unlikely that all COVID-19 deaths are additional, and it is likely that at least some of them replace deaths that would have occurred due to other causes, though the extent to which this is true should not be overstated [8]. There is evidence that during the first wave of the pandemic in spring/summer 2020 there was a slight excess of deaths due to Alzheimer’s and dementia, and circulatory causes, with much larger decreases in respiratory deaths [6]. However, there were lower than expected numbers of deaths for non-COVID-19 reasons during the second wave towards the end of 2020 and into 2021. Given that our results indicate that COVID-19 has had a much larger impact on population health than chronic obstructive pulmonary disease, but a smaller one than ischaemic heart disease, it is unlikely that these sudden changes in competing causes of death would change these conclusions. An excess of deaths due to Alzheimer’s disease may bring DALYs due to Alzheimer’s closer, or slightly higher than our lower limit estimate of COVID-19 DALYs, which again would not substantially alter our conclusions.

Our disease model for health loss due to COVID-19 morbidity is based on a published consensus method outlined by the European Burden of Disease Network and the ECDC [20]. This method can be adapted for both prevalence- or pathogen-based YLD calculations. In our SBOD study, we largely use prevalence-based YLD calculations across our cause list, which means that any future indirect effects from COVID-19 following infection in those with “long COVID”, such as circulatory or respiratory conditions, would be attributed to their relevant non-communicable disease group through prevalence estimates. As such, our range of COVID-19 health states are fully comprehensive based on current evidence, which would not have been the case had we adopted a pathogen-based approach to estimating YLD. However, we acknowledge that estimates of the transition to, and duration of, post-acute consequences remain largely uncertain [27–30]. In our sensitivity analyses, we have shown that a wide range of assumptions have a minimal impact on DALY estimates, given that YLD only contributes around 2% to total DALYs. As further epidemiological information on post-acute consequences emerge, they can be integrated into our estimation process to increase the certainty of our point estimates of YLD. We did not adjust YLD for multimorbidity, which would have resulted in a minimal overestimation of YLD [31].

There has been a debate over how values of YLL are assigned to those who have died. This is centred on the idea that because deaths have mainly occurred in the elderly and frail, including many care home residents, that aspirational or even average values derived from national life tables may be unsuitable [8, 32]. An unrelated issue has also emerged, in that it has been wrongly suggested that since the average age of death of COVID-19 was close to estimates of the age of life expectancy at birth that those people were likely to die soon [33]. This overlooks the fact that remaining life expectancy increases as people survive to older ages, so that estimates of life expectancy at birth do not apply to those who have survived earlier mortality hazards. We have opted to use an aspirational life table for three reasons. Burden of disease studies do not currently use lower life expectancy estimates for deaths that occur in the elderly and frail, for example assigning lower values of life expectancy to individuals with Alzheimer’s disease, so to do so for COVID-19 would be selective, and comparability would be lost [34]. DALYs are a measure of the health gap, rather than a health expectancy, which by definition should be assessed relative to an aspirational counterfactual scenario [35]. Finally, as COVID-19 is a global issue there are great advantages in providing data which is internationally comparable to facilitate reproducible research.

### Comparisons with other published literature

To date, there have been several publications estimating the population health impact of COVID-19 through DALYs, although the application of different methods restricts comparisons between study estimates. The European Burden of Disease Network has been actively collating a repository of relevant journal publications, and grey literature, relating to COVID-19 DALYs [36]. As with any DALY estimates, the need for the application of similar methods dictates the ability to compare end estimates. There have been three other studies published (Germany, Malta, and the Netherlands) for which our estimates can be compared against [37–39]. All other studies have adopted methods which significantly differ from the European Burden of Disease Network’s consensus method, such as using different health states, disability weights, or age-conditional life expectancy weights [20]. Along with our study, the other three studies report low contributions of YLD to DALYs, with the largest contribution reported in Malta (5%). The rate of DALYs in Scotland was the highest, followed by the Netherlands, Malta and Germany [39]. Islam et al. reported a similar ordering, and sense of relative scale in differences between Scotland, the Netherlands and Germany when assessing the impact of the COVID-19 on YLL [40].

Given that death rates were more similar, between Scotland and the Netherlands, than DALY rates, this implies that people in Scotland died of COVID-19 younger than in the Netherlands. This in itself cannot be fully appraised, given that this pattern is true for all-cause YLL too, given than life expectancy in the Netherlands is higher than that of Scotland. Although comparisons in COVID-19 DALYs can be useful to illustrate the extent to which adverse COVID-19 outcomes have been prevented, or mitigated, care must be sought when interpreting them, given the differences in the timing of peaks, and absolute differences in rates might not be the most informative given than baseline levels of vulnerability in each country will significantly differ. Future research should be directed towards international comparisons over longer time periods, incorporating differences in pre-pandemic mortality risks, and utilising methods which are comparable. For those countries, whose aims are not to achieve international comparisons in their primary analysis, supplementary analyses could be carried out, and published to enable several aims to be reached.

### Implications for policy and research

The direct negative impact of COVID-19 on Scottish population health has been substantial. Despite unprecedented mitigation efforts, a novel disease has developed from a single identified case in the early months of spring 2020, to having the second largest population health impact relative to all other health conditions. Unquestionably, the unprecedented extent of mitigation approaches applied to reduce infection and death will have averted significant further damage. Our estimates represent the actual impact on Scotland, rather than the impact had national lockdowns, physical distancing, and other restrictive measures not been implemented.

Burden of disease methodology offers an important opportunity for monitoring both the indirect and direct impacts of the COVID-19 pandemic. As a result of national lockdowns and other restrictions, some factors have improved, such as reductions in some other infection types. However, many have worsened, such as mental health, or the range of conditions impacted by reductions in physical activity. Estimation and attribution of DALYs allows a common lens through which we can frame changes in the population-level health impact of the pandemic on diseases, injuries and risk factors.

Due to the sensitive, and political, nature of the response to the pandemic, we advise that countries calculate their own estimates of COVID-19 DALYs where they hold relevant data, so that the inputs and methods are fully understood. If estimates are sourced from future iterations of the GBD study, users must ensure that the inputs and outputs are representative of their country-specific understanding of the occurrence, and severity, of infection, and the number of deaths [41].

In December 2020, vaccination to tackle COVID-19 commenced across the UK [42]. Safe and effective vaccines will be the most powerful tool to tackle COVID-19 infection, although non-pharmaceutical interventions may still be required. Periodic estimation of DALYs during 2021 and beyond is required. This, alongside international comparisons, will provide indications of the fuller impact of COVID-19, and will provide insights into the scale of DALYs, which are being averted due to the national rollout of the vaccination programme, and other continued mitigation efforts. Furthermore, it is essential for periodic estimation as the emergence of new variants, with at least increased transmissibility, and their uncertainty may threaten our attempts to control infection.

Many studies have indicated that, on average, people suffering from adverse outcomes and death have had many prior co-morbidities. Although risk-outcome relationships have not yet been developed, there is descriptive evidence that people with prior exposure to metabolic (such as obesity) and behavioural (such as tobacco smoking) risks, or with pre-existing co-morbidities linked to these risks, were more likely to suffer from poorer outcomes from infection [43, 44]. As such, we would expect a high proportion of COVID-19 DALYs, and YLL, to be attributable to prior risk factors.

As the indirect harms, following attempts to control the COVID-19 pandemic, continue to accumulate, there is an increasing need for rapid preventative action to recover and improve population health and reduce inequalities in population health in Scotland [45]. Prior to the pandemic, improvements in population health in Scotland had faltered since 2012, with a slowdown in the overall progress of reducing mortality and widening of socioeconomic inequalities in mortality [46–48]. Proactively building a healthier Scotland with lower levels and milder forms of non-communicable disease will act as an important barrier in reducing any direct or indirect harms from future epidemics or disasters [49–51].

## Supplementary Information

**Additional file 1:.** Daily COVID-19 infection data by health state for Scotland, 2020

**Additional file 2: Table S1** SBOD estimates of the number of DALYs for the 15 leading causes of disease and injury, Scotland, 2018

**Additional file 3: Table S2** Morbidity sensitivity analyses: impact on COVID-19 YLD and DALYs, Scotland, 2020

**Additional file 4: Table S3** GBD 2019 estimates of the number of DALYs for the 15 leading causes of disease and injury, Scotland, 2019

## Data Availability

Summary data from the datasets supporting the conclusions of this article that were used to estimate COVID-19 morbidity in Scotland are available online in the Supplementary Information section. The underlying mortality data that was used in intermediate calculations for COVID-19 YLL estimates are not publically available, although summary data by aggregated age-groups are available from the website of the National Records of Scotland (https://www.nrscotland.gov.uk/). Requests for this data can be made to National Records of Scotland, Information Governance Team, Ladywell House, Ladywell Road, Edinburgh, EH12 7TF (https://www.nrscotland.gov.uk/about-us/contact-us).

## Abbreviations

COVID-19: Coronavirus Disease 2019
DALYs: Disability-Adjusted Life Years
ECDC: European Centre for Disease Prevention and Control
EDWS: European Disability Weight Study
GBD: Global Burden of Disease
GHDx: Global Health Data Exchange
ICD: International Classification of Diseases
ICU: Intensive Care Unit
NRS: National Records of Scotland
PHEIC: Public Health Emergency of International Concern
PHS: Public Health Scotland
QALYs: Quality-Adjusted Life Years
SBOD: Scottish Burden of Disease
SEIR: Susceptible → Exposed → Infected → Recovered
UK: United Kingdom
WHO: World Health Organization
YLD: Years Lived with Disability
YLL: Years of Life Lost to premature mortality
