# Supplementary material for: Measuring disability-adjusted life years (DALYs) due to COVID-19 in Scotland, 2020
Source: Arch Public Health. 2022 Apr 1;80:105. doi: 10.1186/s13690-022-00862-x (PMC8972687; doi:10.1186/s13690-022-00862-x)
Supplement: Supplementary file 2 — Additional file 2: Table S1 SBOD estimates of the number of DALYs for the 15 leading causes of disease and injury, Scotland, 2018 [file 13690_2022_862_MOESM2_ESM.docx]

**Table S1. SBOD estimates of the number of DALYs for the 15 leading causes of disease and injury, Scotland, 2018**

| **Cause of disease or injury** | **Ranking of DALYs** | **Number of DALYs** |
| --- | --- | --- |
| Ischaemic heart disease | 1 | 138,189 |
| Alzheimer's and other dementias | 2 | 90,695 |
| Lung cancer | 3 | 85,357 |
| Drug use disorders | 4 | 80,855 |
| Cerebrovascular disease | 5 | 76,810 |
| Chronic obstructive pulmonary disease | 6 | 72,024 |
| Low back and neck pain | 7 | 68,100 |
| Depression | 8 | 67,171 |
| Migraine | 9 | 47,860 |
| Anxiety disorders | 10 | 46,134 |
| Sense organ diseases | 11 | 42,670 |
| Colorectal cancer | 12 | 41,704 |
| Diabetes | 13 | 40,018 |
| Lower respiratory infections | 14 | 39,233 |
| Suicide and self-harm related injuries | 15 | 37,412 |

‘SBOD’ denotes Scottish Burden of Disease study; ‘DALYs’ denotes disability-adjusted life years.
