# Supplementary material for: Measuring disability-adjusted life years (DALYs) due to COVID-19 in Scotland, 2020
Source: Arch Public Health. 2022 Apr 1;80:105. doi: 10.1186/s13690-022-00862-x (PMC8972687; doi:10.1186/s13690-022-00862-x)
Supplement: Supplementary file 3 — Additional file 3: Table S2 Morbidity sensitivity analyses: impact on COVID-19 YLD and DALYs, Scotland, 2020 [file 13690_2022_862_MOESM3_ESM.docx]

**Table S2. Morbidity sensitivity analyses: impact on COVID-19 YLD and DALYs, Scotland, 2020**

| **Scenario** | **Impacted health states** | **Sensitivity** | **Total YLD** | **Total DALYs** |
| --- | --- | --- | --- | --- |
| Community cases | Moderate | Increase moderate cases by 10% | 1,920 | 96,553 – 108,277 |
|  |  | Increase moderate cases by 25% | 1,971 | 96,604 – 108,328 |
|  |  | Increase moderate cases by 50% | 2,057 | 96,689 – 108,414 |
| Post-acute consequences | Transition to post-acute consequences for all cases | Double transition probability | 3,319 | 97,952 – 109,676 |
|  |  | Half transition probability | 1,170 | 95,803 – 107,527 |
|  |  | Double the duration | 3,319 | 97,952 – 109,676 |
|  |  | Half the duration | 1,170 | 95,803 – 107,527 |
|  | Transition to post-acute consequences for symptomatic cases | Apply transition probability used in main estimate | 1,170 | 95,803 – 107,527 |
|  |  | Double transition probability | 1,886 | 96,519 – 108,243 |
|  |  | Half transition probability | 811 | 95,444 – 107,169 |
|  |  | Double the duration | 1,886 | 96,519 – 108,243 |
|  |  | Half the duration | 811 | 95,444 – 107,169 |
| **Combination of scenarios that minimise YLD impact ^a^** | | | **632** | **95,265 – 106,989** |
| **Combination of scenarios that maximise YLD impact ^b^** | | | **6,356** | **100,989 – 112,713** |

a Combined criteria used: no increase in moderate cases; transition to post-acute consequences for symptomatic cases only; half transition of acute to post-acute consequences; half the duration for post-acute consequences.

b Combined criteria used: increase moderate cases by 50%; double transition of acute to post-acute consequences; double the duration for post-acute consequences.
