# Supplementary material for: Measuring disability-adjusted life years (DALYs) due to COVID-19 in Scotland, 2020
Source: Arch Public Health. 2022 Apr 1;80:105. doi: 10.1186/s13690-022-00862-x (PMC8972687; doi:10.1186/s13690-022-00862-x)
Supplement: Supplementary file 4 — Additional file 4: Table S3 GBD 2019 estimates of the number of DALYs for the 15 leading causes of disease and injury, Scotland, 2019 [file 13690_2022_862_MOESM4_ESM.docx]

**Table S3. GBD 2019 estimates of the number of DALYs for the 15 leading causes of disease and injury, Scotland, 2019**

| **Cause of disease or injury** | **Ranking of DALYs** | **Number of DALYs** |
| --- | --- | --- |
| Ischemic heart disease | 1 | 158,257 |
| Chronic obstructive pulmonary disease | 2 | 94,190 |
| Stroke | 3 | 92,767 |
| Tracheal, bronchus, and lung cancer | 4 | 91,381 |
| Low back pain | 5 | 81,697 |
| Diabetes mellitus | 6 | 57,923 |
| Colon and rectum cancer | 7 | 52,804 |
| Falls | 8 | 50,658 |
| Drug use disorders | 9 | 50,293 |
| Headache disorders | 10 | 44,886 |
| Depressive disorders | 11 | 43,594 |
| Lower respiratory infections | 12 | 38,163 |
| Alzheimer's disease and other dementias | 13 | 37,090 |
| Cirrhosis and other chronic liver diseases | 14 | 34,614 |
| Gynecological diseases | 15 | 32,015 |

‘DALYs’ denotes disability-adjusted life years; GBD Compare query used to generate data: http://ihmeuw.org/5f2r
